# Assessment of the Abbott BinaxNOW SARS-CoV-2 rapid antigen test against viral variants of concern

**DOI:** 10.1016/j.isci.2022.103968

**Published:** 2022-02-22

**Authors:** Anuradha Rao, Leda Bassit, Jessica Lin, Kiran Verma, Heather B. Bowers, Kimberly Pachura, Morgan Greenleaf, Julie Sullivan, Eric Lai, Richard S. Creager, Thomas Pribyl, John Blackwood, Anne L. Piantadosi, Raymond Schinazi, Greg S. Martin, Wilbur A. Lam

**Affiliations:** 1 Emory University School of Medicine, Emory University, Room 448, Atlanta, GA 30322, USA; 2 Georgia Institute of Technology, Atlanta, GA 30332, USA; 3 Personalized Science, LLC, Burlington, VT, 05403; 4 NaviDx, LLC, Newport Beach, CA 92660; 5 BiocomX, Dana Point, CA 92629, USA

**Keywords:** Biological sciences, Biotechnology, Diagnostics, Public health, Virology

## Abstract

As the emergence of SARS-CoV-2 variants brings the global pandemic to new levels, the performance of current rapid antigen tests against variants of concern and interest (VOC/I) is of significant public health concern. Here, we report assessment of the Abbot BinaxNOW COVID-19 Antigen Self-Test. Using genetically sequenced remnant clinical samples collected from individuals positive for SARS-CoV-2, we assessed the performance of BinaxNOW against the variants that currently pose public health threats. We measured the limit of detection of BinaxNOW against various VOC/I in a blinded manner. BinaxNOW successfully detected the Omicron (B.1.1.529), Mu (B.1.621), Delta (B.1.617.2), Lambda (C.37), Gamma (P.1), Alpha (B.1.1.7), Beta (B.1.351), Eta (B.1.525), and P.2 variants and at low viral concentrations. BinaxNOW also detected the Omicron variant in individual remnant clinical samples. Overall, these data indicate that this inexpensive and simple-to-use, FDA-authorized and broadly distributed rapid test can reliably detect Omicron, Delta, and other VOC/I.

## Highlights

• Pooled remnant clinical samples with variants of concern are tested using BinaxNOW
• BinaxNOW detects live and heat-inactivated virus in pooled and individual samples
• BinaxNOW sensitivity slightly lower against Omicron variant in laboratory tests
• BinaxNOW remains a useful public health tool to combat the COVID-19 pandemic

## Graphical Abstract

**Figure:**
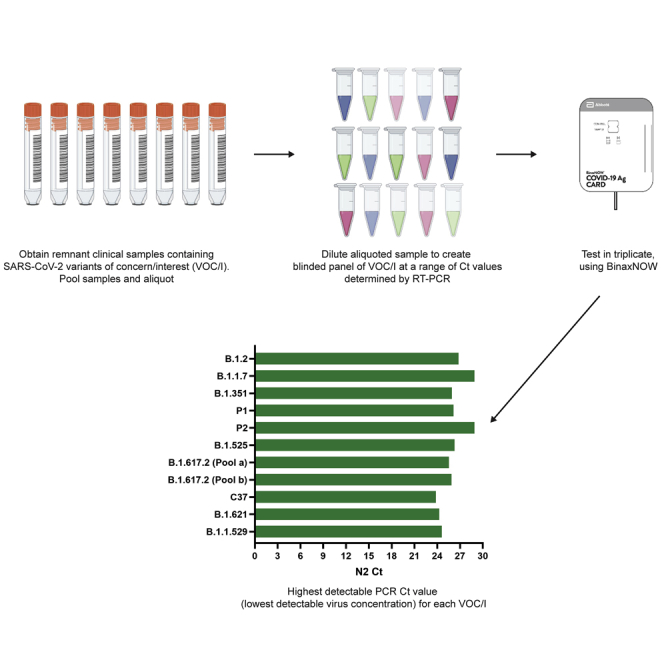

## Introduction

The emergence of new SARS-CoV-2 variants has pushed the pandemic to new levels, raising concerns of increased infectivity, breakthrough cases among vaccinated individuals, and the viability of current test strategies. In 2021, new SARS-CoV-2 variants of concern and interest (VOC/I) such as the Delta (B.1.617.2), Lambda (C37), Mu (B.1.621), and Omicron (B.1.1.529) variants have caused multiple surges of COVID-19 cases worldwide and raised concerns about evasion of the currently available SARS-CoV-2 vaccines (Herlihy, 2021; Lopez Bernal et al., 2021; Shah et al., 2021b; Tchesnokova et al., 2021).

With the high prevalence of rapid diagnostic assays currently available to the public in point-of-care (POC) settings and as at-home over-the-counter (OTC) kits, an obvious question is whether these tests can even reliably detect Delta, Omicron, and other variants of concern/interest (VOC/I). The timing of detection is particularly important for Omicron, as it has been shown to have a shorter incubation period than previous variants, and thus may become transmissible faster after infection (Brandal et al., 2021; Burki, 2021; Jansen, 2021). If these home and community-based tests can indeed detect VOC/I, they can be implemented as part of broad public health strategies to help curtail the rapid spread of VOC/I. On the other hand, if these rapid tests cannot reliably detect the most prevalent VOC/I, their overall clinical utility at the current point of the pandemic should be called into question.

To that end, here, we report our objective assessment of Abbott’s BinaxNOW COVID-19 Antigen Self-Test, which has among the highest usage, availability, distribution, and production rates of rapid tests and was the first lateral flow assay (LFA)-based rapid antigen test to receive U.S. FDA Emergency Use Authorization (EUA) for the home OTC setting (Shah et al., 2021a; Prince-Guerra, 2021; Pollock et al., 2021; Hodges, 2021). BinaxNOW is a SARS-CoV-2 diagnostic assay that detects the viral nucleocapsid (N) protein in samples collected by anterior nasal swab and reports a qualitative positive, negative, or invalid result (BINAXNOW COVID-19 AG CARD, n,d). We previously assessed the usability of BinaxNOW as a self-administered test and conducted initial experiments assessing the test’s performance in detecting wild type virus and various variants (Frediani et al., 2021). Here, we report with a higher molecular resolution on the performance of BinaxNOW using a comprehensive panel of VOC/I that is currently of the highest public health significance.

**Figure 1:**
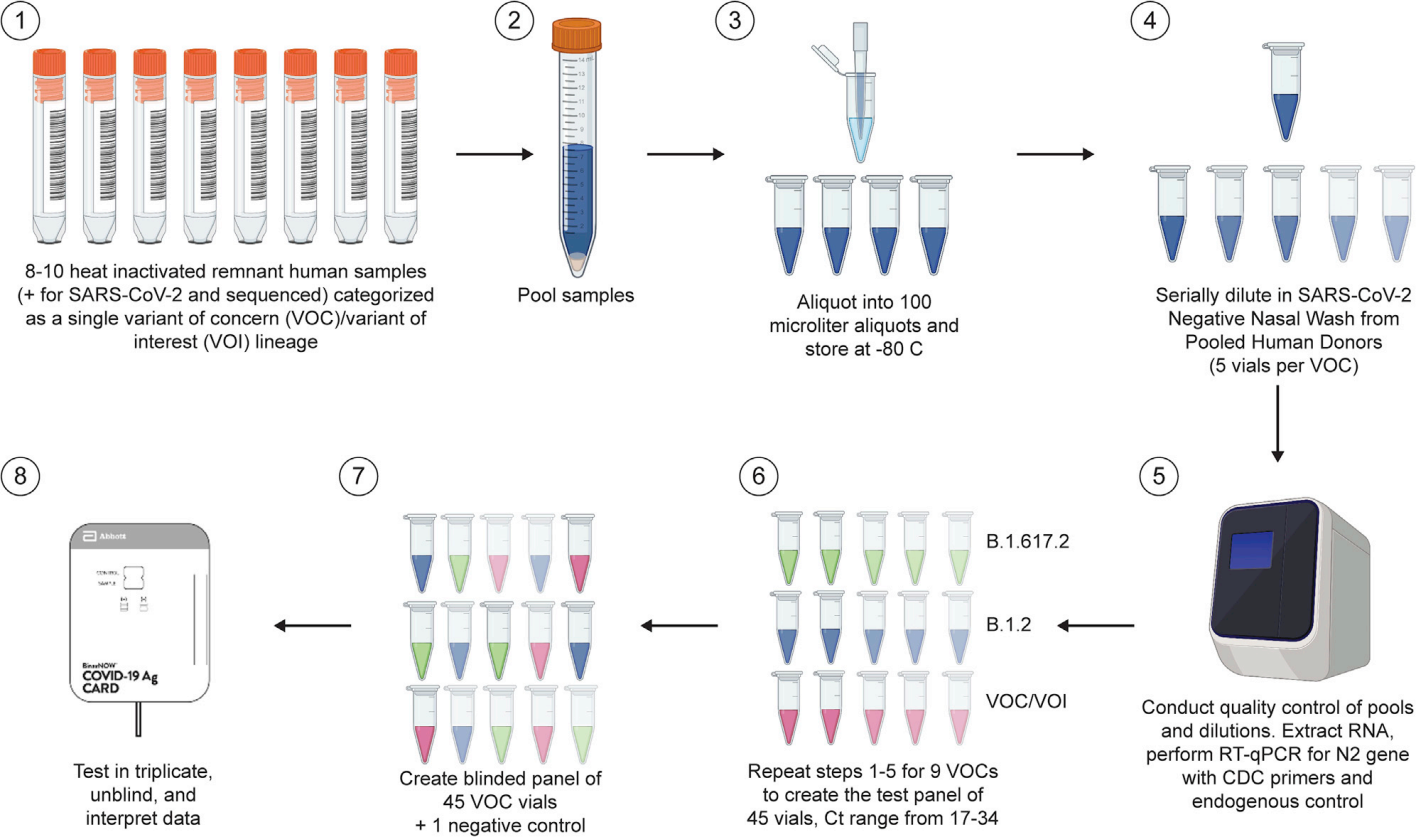
Summary of the procedures implemented for SARS-CoV-2 remnant clinical sample pooling, creation of variant panels and panel dilutions, and assessment of the Abbot BinaxNOW COVID-19 Antigen Self-Test against the current viral variants of concern (VOC) and variants of interest (VOI) Pooled remnant clinical samples were diluted in SARS-CoV-2 negative nasal wash, and viral load was measured by RT-qPCR, following which the performance of BinaxNOW was assessed in a blinded manner and triplicate. (N2: nucleocapsid).

In September 2021, the Mu (B.1.621) variant had been detected in every state in the US and was designated as a VOC by the World Health Organization (WHO), though it was later reclassified as a VOI (World Health Organization, 2022). Importantly, the Mu variant harbors a mutation, E484K, that likely enables the virus to blunt vaccine and infection-induced immunity (Wadman, 2021). Given the potential at the time for this variant to significantly worsen the outlook of the global pandemic, we tested the performance of BinaxNOW in duplicate and a blinded manner using remnant clinical samples (RCS) (Figure 1).

Most recently, the Omicron variant (B.1.1.529) has emerged and been designated a VOC by the WHO due to a high number of mutations in the spike protein (World Health Organization, 2021). The Omicron variant has 32 additional mutations in the spike protein, which may allow it to partially evade vaccine-elicited immunity by escaping neutralizing antibodies from previous strains of SARS-CoV-2 (Cao et al., 2021; Cele et al., 2021; Liu et al., 2021). Owing to its increased transmissibility, Omicron has quickly become the dominant variant in the United States and around the world (CDC, 2020). For these reasons, we assessed the performance of BinaxNOW using pooled, heat-inactivated RCS positive for Omicron (B.1.1.529) variant.

## Results

Overall, as detailed in Table 1, the BinaxNOW successfully detected the Mu (B.1.621) variant in individual RCS with Ct values ranging from 23 (highest viral concentration) to 30.3 (lowest viral concentration), which is within the clinically relevant range reported by other groups (Faíco-Filho et al., 2020; Jaafar et al., 2021; Magleby et al., 2020). BinaxNOW also successfully detected B.1.2 in individual RCS with Ct values ranging from 22 (highest viral concentration) to 34.8 (lowest viral concentration).

**Table 1:** Assessment of the Abbot BinaxNOW COVID-19 Antigen Self-Test against the Mu (B.1.621) viral variant in genetically sequenced clinical nasal swab samples

Sample ID	Lineage	Dilution factor	N2 Ct	N2 Ct Std Dev	Replicate 1	Replicate 2	Final Result
					Result +/−	Result +/−	
Sample 1	B.1.2		22.0	0.01	Positive	Positive	Positive
Sample 2	B.1.2		22.7	0.16	Positive	Positive	Positive
Sample 3	B.1.2		24.3	0.10	Positive	Positive	Positive
Sample 4	B.1.2		20.1	0.13	Positive	Positive	Positive
Sample 5	B.1.2		27.7	0.08	Positive	Positive	Positive
Sample 6	B.1.2		22.9	0.05	Positive	Positive	Positive
Sample 7	B.1.2		32.4	0.12	Positive	Positive	Positive
Sample 8	B.1.2		34.8	0.91	Positive	Positive	Positive
Sample 8	B.1.2	4	35.4	0.58	Negative	Negative	Negative
Sample 8	B.1.2	16	36.5	0.80	Negative	Negative	Negative
Sample 8	B.1.2	64	0	–	Negative	Negative	Negative
Sample 8	B.1.2	256	0	–	Negative	Negative	Negative
Sample 8	B.1.2	1024	0	–	Negative	Negative	Negative
Sample 8	B.1.2	4096	0	–	Negative	Negative	Negative
Sample 8	B.1.2	16,384	0	–	Negative	Negative	Negative
Sample 8	B.1.2	65,536	0	–	Negative	Negative	Negative
Sample 9	B.1.621		24.3	0.23	Positive	Positive	Positive
Sample 10	B.1.621		28.5	0.06	Positive	Positive	Positive
Sample 11	B.1.621		23	0.15	Positive	Positive	Positive
Sample 12	B.1.621		24	0.03	Positive	Positive	Positive
Sample 13	B.1.621		26.7	0.21	Positive	Positive	Positive
Sample 14	B.1.621		27	0.15	Positive	Positive	Positive
Sample 15	B.1.621		26.6	0.03	Positive	Positive	Positive
Sample 16	B.1.621		28.9	0.08	Positive	Positive	Positive
Sample 16	B.1.621	4	28.2	0.03	Positive	Positive	Positive
Sample 16	B.1.621	16	30.3	0.02	Positive	Positive	Positive
Sample 16	B.1.621	64	33.1	0.06	Negative	Negative	Negative
Sample 16	B.1.621	256	34.6	0.42	Negative	Negative	Negative
Sample 16	B.1.621	1024	37.3	0.68	Negative	Negative	Negative
Sample 16	B.1.621	4096	37.3	1.12	Negative	Negative	Negative
Sample 16	B.1.621	16,384	0	–	Negative	Negative	Negative
Sample 16	B.1.621	65,536	0	–	Negative	Negative	Negative
Sample 17	Negative control		0	–	Negative	Negative	Negative
Sample 18	Negative control		0	–	Negative	Negative	Negative

RT-qPCR for the SARS-CoV-2 N2 gene using CDC primers/probe set was also performed on each RCS sample and cycle threshold (Ct) values were used as estimates of viral load. For comparison, samples one to eight were obtained from individuals infected with B.1.2, whereas samples 9 to 16 were obtained from RCS positive for Mu (B.1.621) variant. Samples 8 and 16 were also serially diluted and again assessed with RT-qPCR, to assess the limit of detection of the assay.

**Figure 2:**
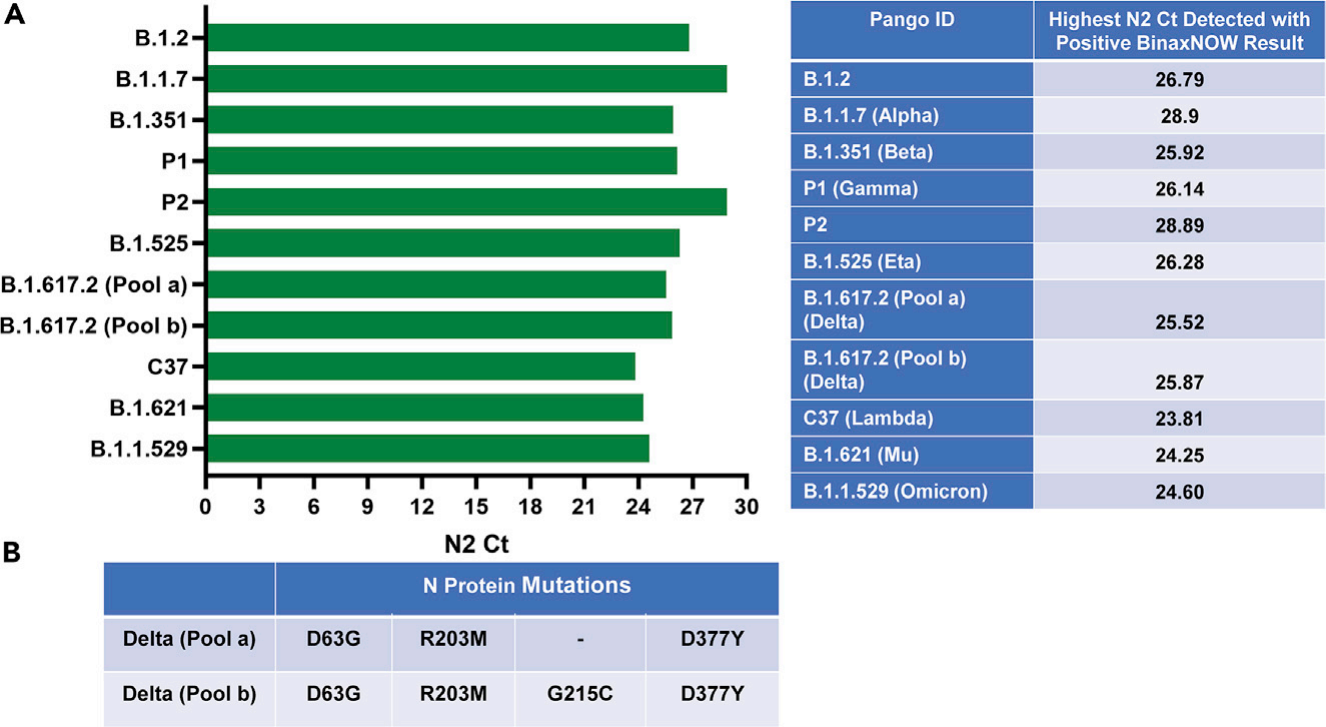
Limit of detection of the Abbott BinaxNOW COVID-19 Antigen Self-Test against the current SARS-CoV-2 variants of concern and interest using pooled heat-inactivated samples (A) Lowest viral load (highest RT-qPCR Ct value) for the indicated VOC/I detected by the BinaxNOW SARS-CoV-2 antigen assay. Green bars indicate successful detection (defined as within three Ct values) of the specific VOC/I compared to that of the control, the SARS-CoV-2 variant B.1.2 (top bar), which is not considered to be a VOC/I. (B) Different nucleocapsid (N) protein mutations in the indicated Delta VOC pools.

BinaxNOW successfully detected Mu (B.1.621), Delta (B.1.617.2), Lambda (C37), Gamma (P1), Alpha (B.1.1.7), Beta (1.351), Eta (B.1.525), and P2 viral variants in RCS pools consistently from samples with the lowest Ct values of 23.8, (highest viral concentrations) to those with the highest detectable Ct values of 28.9, (lowest viral concentrations) (Figure 2), which is within the clinically relevant range reported by other groups. Moreover, BinaxNOW also successfully detected subtypes of Delta (B.1.617.2), which harbor different N protein mutations. Our results also showed that BinaxNOW successfully detected all the aforementioned VOC/I with equivalent sensitivity, defined as within three Ct values of B.1.2.

**Figure 3:**
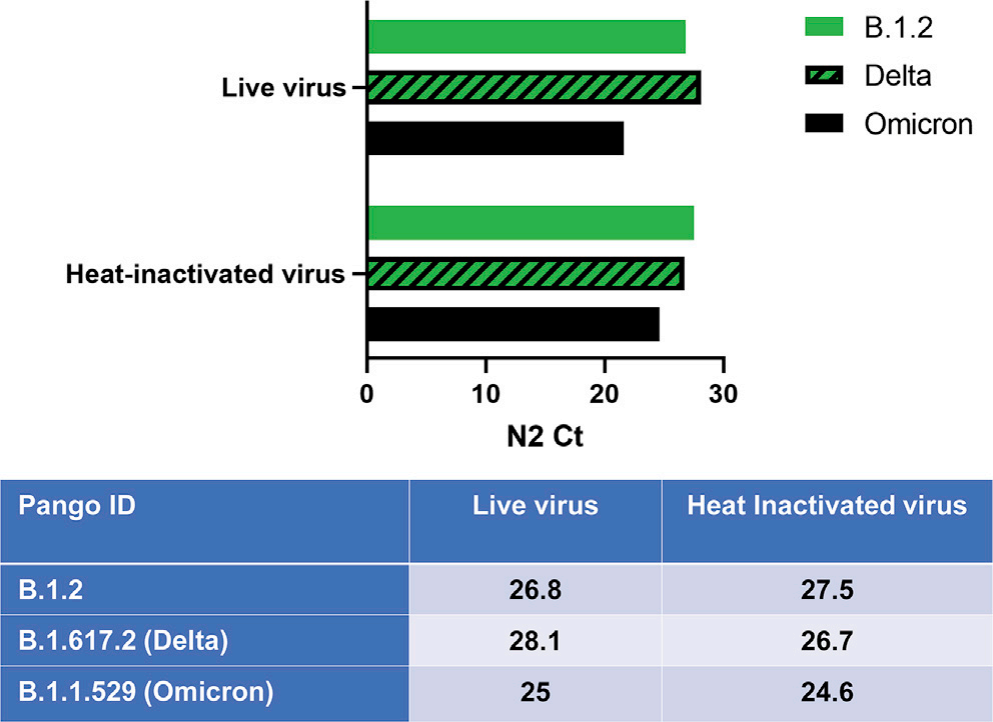
Performance of the Abbot BinaxNOW COVID-19 Antigen Self-Test against live Omicron (B.1.1.529) variant The highest detectable Ct value (lowest detectable viral load) of the BinaxNOW against the Omicron variant, Delta variant, and a control are shown. Performance was assessed against both live virus and heat-inactivated virus.

BinaxNOW successfully and consistently detected the Omicron (B.1.1.529) variant in heat-inactivated pooled clinical samples with the highest detected Ct value of 24.6 (Figure 2). This value is lower than the highest detected value of other variants of concern, which may indicate that the BinaxNOW has lower sensitivity against the Omicron (B.1.1.529) variant compared to the previous VOC (Figure 2). BinaxNOW also demonstrated a lower sensitivity against pooled live Omicron (B.1.1.529) RCS compared to B.1.2 and Delta (B.1.617.2) RCS. The highest detected Ct value for Omicron (B.1.1.529) was 25, which is lower than that detected for B.1.2 (Ct 26.8) and Delta (B.1.617.2) (Ct 28.1) (Figure 3). In individual RCS, BinaxNOW detected samples with N2 Ct values as high as 28.3 (Table 2). The highest detectable Ct value of the individual Omicron samples is lower than that of previously tested individual control (B.1.2) samples and samples containing the Mu (B.1.621) variant (Table 1).

**Table 2:** Assessment of the Abbot BinaxNOW COVID-19 Antigen Self-Test against individual patient samples containing the Omicron (B.1.1.529) variant

Sample	Lineage	Avg N2 Ct (3 replicates	Std Dev N2 Ct	Replicate 1	Replicate2	Final Result
Sample 1	B.1.1.529	21.2	0.15	Positive	Positive	Positive
Sample 6	B.1.1.529	24.7	0.05	Positive	Positive	Positive
Sample 3	B.1.1.529	25.8	0.04	Positive	Positive	Positive
Sample 4	B.1.1.529	26.2	0.05	Positive	Positive	Positive
Sample 7	B.1.1.529	26.3	0.11	Positive	Positive	Positive
Sample 9	B.1.1.529	26.3	0.08	Positive	Positive	Positive
Sample 2	B.1.1.529	27	0.06	Positive	Positive	Positive
Sample 8	B.1.1.529	28.2	0.15	Positive	Positive	Positive
Sample 10	B.1.1.529	28.2	0.14	Positive	Positive	Positive
Sample 12	B.1.1.529	28.2	0.29	Positive	Positive	Positive
Sample 5	B.1.1.529	28.3	0.11	Positive	Positive	Positive
Sample 11	B.1.1.529	29.4	0.16	Negative	Negative	Negative

Genetically sequenced remnant clinical samples (N = 12) containing the N:D343G mutation were measured in triplicate using RT-qPCR, as a proxy for viral load. The samples were then tested in duplicate using the BinaxNOW COVID-19 Antigen Self-Test. The sample with the highest detectable Ct is highlighted in green.

## Discussion

Although the sensitivity of BinaxNOW appears to be slightly decreased against the Omicron (B.1.1.529) variant compared to previous VOC/I, the results still justify the continued use of this readily available, inexpensive, and simple-to-use rapid test kit as part of the community- and/or home-based testing strategies to combat the ongoing public health crisis. Ultimately, laboratory experiments cannot fully recapitulate the real-world application of a test kit, and the utility of the BinaxNOW will depend on a review of its clinical performance, which we are currently conducting.

### Limitations of the study

We acknowledge the limitations of the current study are that we used RCS that may have some degradation and may not accurately reflect real-world testing conditions. In addition, the BinaxNOW kit lots used in this study varied from experiment to experiment and that could have generated slight differences in the test performance. Additional studies into the quantitative differences in the N antigen levels of Omicron variant patient samples will help to clarify the implications of the decreased performance. Future studies are also underway to compare BinaxNOW to other available rapid antigen tests.

## STAR★Methods

### Key resources table

**Table:** 

REAGENT or RESOURCE	SOURCE	IDENTIFIER
**Biological samples**		

Remnant Clinical Samples containing SARS-CoV-2 variants Mu (B.1.621) Delta (B.1.617.2), Lambda (C37), and Gamma (P1) and B.1.2	Helix OpCo, LLC	N/A
Remnant clinical samples containing SARS-CoV-2 Omicron (B.1.1.529)	University of Washington	N/A

**Critical commercial assays**		

CDC 2019-nCoV RT-PCR Diagnostic Panel	IDT	Catalog # 10006770
MagMax Viral RNA Isolation Kit	Applied Biosystems	Catalog # AM1939
qScript XLT 1-Step RT-qPCR ToughMix	QuantaBio	Catalog # 95133-100
BinaxNOW COVID-19 Antigen Self-Test	Abbott	N/A

**Software and algorithms**		

Rosalind Diagnostic Monitoring (DxM) system	RadX	https://radx.rosalind.bio
GraphPad Prism	GraphPad	https://www.graphpad.com/scientific-software/prism/
NextClade	NextClade	https://clades.nextstrain.org/

**Other**		

Negative Nasal Wash	Lee BioSolutions	Catalog # 991-26-P
KingFisher Apex System	Thermo Fisher Scientific	Catalog # 5400910
LightCycler 480II	Roche	Catalog # 05015278001

### Resource availability

#### Lead contact

Requests for further information and resources should be directed to the lead contact Wilbur Lam (wilbur.lam@emory.edu), or the co-corresponding author Greg Martin (greg.martin@emory.edu).

#### Materials availability

This study did not generate new materials.

### Experimental model and subject details

The study abides by the ethical guidelines of research at Emory University. The de-identified remnant clinical samples in this study were provided without any patient information. Sequencing of the SARS-CoV-2 to confirm the variant was done by the party from which we obtained the remnant clinical samples.

### Method details

Heat-inactivated RCS (provided by Helix OpCo, LLC) from individuals positive for SARS-CoV-2 (N = 8) known to be infected with the Mu variant were selected based on genomic sequence quality (as determined by NextClade) and cycle threshold (Ct) value of N protein. We compared these results to samples obtained from individuals (N = 8) infected with the B.1.2 strain of SARS-CoV-2, which is not considered to be a VOC/I by the WHO, and therefore served as our comparator. RT-qPCR for the SARS-CoV-2 N2 gene using CDC primers/probe set was performed on each RCS and N2 Ct values were used as estimates of viral load.

In addition, using genetically sequenced RCS collected from individuals positive for SARS-CoV-2 across the country, we created RCS “pools” of each of the VOC/I (Figure 1). The individual VOC/I pools were verified by repeating genetic sequencing to ensure quality control. Panels of the VOC/I pools of varying viral loads were then created by serial dilution using SARS-CoV-2 negative pooled human donor nasal wash (Lee Biosolutions, Catalog No. 991-26-P-1). Dilutions of every pool were then analyzed by RT-qPCR for the SARS-CoV-2 N2 gene using CDC primers/probe set (Figure 1). The performance of BinaxNOW was then assessed in a blinded manner in triplicate.

The pools for heat-inactivated Omicron variant were created using the same method as previously stated (Figure 1) and diluted to 10 dilutions that ranged from a Ct value of 21.2 to a Ct value of 31.7. The limit of detection was determined by testing each sample dilution 5 times with BinaxNOW. For testing, we used the direct spike method, where 20μL of sample was spiked onto the swab provided with the test and subsequent steps were according to the BinaxNOW instructions for use (IFU). This limit of detection was further confirmed by testing the highest detectable dilution as well as the two neighboring dilutions a further 20 times.

We then assessed BinaxNOW using non heat inactivated RCS obtained from the University of Washington that were confirmed to be Omicron (B.1.1.529). These samples were pooled and diluted to a range of Ct values between 19.3 and 28.8. The dilutions were tested according to the IFU of BinaxNOW rapid antigen test to determine the limit of detection. Finally, we obtained 12 RCS sequence confirmed to be Omicron from LabCorp and measured N2 Ct in triplicate by RT-qPCR (Table 2). These samples were tested in duplicate using BinaxNOW, following the IFU.

### Quantification and statistical analysis

The mean and standard deviation of the Ct values in Tables 1 and 2 were calculated using Microsoft Excel.

## Data Availability

The genetic sequence data was analyzed the Nextclade (https://clades.nextstrain.org/) version 1.7.1 or higher. SARS-CoV-2 strains to be tested were identified using the Rosalind (https://radx.rosalind.bio) bioinformatics platform, version 3.33.1.0 or higher. Any additional information required to reanalyze the data reported in this paper is available from the lead contact upon request
